# Effect of Kernel Size and Its Potential Interaction with Genotype on Key Quality Traits of Durum Wheat

**DOI:** 10.3390/foods10122992

**Published:** 2021-12-04

**Authors:** Kun Wang, Dale Taylor, Yuming Chen, Jerry Suchy, Bin Xiao Fu

**Affiliations:** Grain Research Laboratory, Canadian Grain Commission, 303 Main Street, Winnipeg, MB R3C 3G8, Canada; kun.wang@grainscanada.gc.ca (K.W.); dale.taylor@grainscanada.gc.ca (D.T.); yuming.chen@grainscanada.gc.ca (Y.C.); jerry.suchy@grainscanada.gc.ca (J.S.)

**Keywords:** durum wheat, kernel size, genotype, milling quality, semolina quality, pasta color

## Abstract

This study was conducted to evaluate the influence of kernel size and its potential interaction with genotype on durum wheat quality with emphases on kernel physical characteristics, milling performance, and color-related quality parameters. Wheat samples of seven genotypes, selected from the 2018 Canadian durum variety registration trial, were segregated into large (LK), medium (MK), and small-sized kernels (SK). In general, the kernel size greatly affected the durum wheat milling performance. Within a given size fraction, a strong impact of genotype was shown on the test weight of SK and the milling yields of MK and LK. Particularly, the MK fraction, segregated from the genotypes with superior milling quality, had a higher semolina yield than LK from the genotypes of inferior milling quality, inferring the importance of intrinsic physicochemical properties of durum kernels in affecting milling quality. SK exhibited inferior milling quality regardless of the genotypes selected. A strong impact of genotype was shown for the total yellow pigment (TYP) content and yellowness of semolina, while the kernel size had a significant impact on the brightness and redness of the semolina and pasta. Despite SK possessing much higher TYP, the semolina and pasta prepared from SK were lower in brightness and yellowness but with elevated redness.

## 1. Introduction

Durum wheat physical properties are very important in determining its commercial value. Strong associations have been reported between kernel physical characteristics and durum wheat milling performance, semolina composition, and pasta processing quality [1,2,3,4,5,6]. Emphasis has been on unveiling the relationship between test weight (TWT) to durum wheat milling potential by evaluating samples with a wide range of TWT, protein content, and kernel size distribution (KSD) [3,4,5]. Recent study in our laboratory has shown that kernel size is more effective than TWT in predicting the milling performance of durum wheat by assessing Canadian durum samples with a wide range of TWT and KSD [5].

In general, with the decrease of kernel size from large to medium, the semolina and total milling yields of durum wheat reduced gradually. A drastic decrease in milling quality was observed for small kernels passing through the no. 6 slotted sieve (2.38 mm aperture) [4,5] with much reduced milling yields coupled with elevated ash content. Baasandorj, Ohm, Manthey, and Simsek (2015) studied the impact of kernel size and mill type on the milling and baking quality of hard red spring wheat [7]. Compared with large-sized kernels, the small-sized kernels had a much lower flour yield because of the lower proportion of starchy endosperm to bran.

The kernel size of durum wheat can significantly affect not only the milling performance but also the semolina and pasta quality [3,5]. Semolina milled from SK exhibited higher protein content, finer granulation, and was higher in TYP but less bright in color with elevated ash [3,5]. Cooked pasta made from durum samples with a high proportion of SK had higher firmness but was duller in color.

While the impact of kernel size on semolina and pasta quality is well-documented, limited information is available on the response of genotype to the general relationships between kernel size and the key durum wheat quality parameters. Due to the variation in intrinsic quality, the degree of impact of kernel size on quality could be genotype dependent. Using milling performance as an example, it is not clear if the genotypes with superior milling quality would be less susceptible to kernel size variation than those of inferior milling quality, or vice versa. Genotypes with different intrinsic quality could respond differently to variations in kernel size.

On the other hand, differences in quality among genotypes could be affected by the variation in kernel size. Although TKW was shown to be highly correlated with semolina yield across four different durum varieties (*R*^2^ = 0.92) evaluated by Wang and Fu (2020), greater variation in semolina yield was seen for larger kernels than for smaller ones [5]. The fact that the genotypic variation in durum milling performance was related to kernel size suggests a potentially greater role of genotype in the milling quality of large kernels than that of the small ones.

With the prevalence of hot and dry growing conditions on Canadian prairies in the last few years, some durum samples, although graded as No.1 or No. 2 Canada Western Amber Durum (CWAD), showed relatively wide range of KSD and milling quality [5]. To optimize the commercial value of durum wheat of different KSD and understand how quality parameters respond to kernel size variations, a thorough investigation is required to further elucidate the combined effect of kernel size and genotype on key durum wheat quality parameters.

Therefore, the objective of this study was to evaluate the influence of kernel size and its potential interaction with genotype on key durum wheat quality traits with emphases on the wheat physical properties, milling performance, and color-related quality attributes.

## 2. Materials and Methods

### 2.1. Wheat Samples

Seven genotypes were selected from the 2018 Canadian durum wheat variety registration trial based on their intrinsic differences in milling and color-related quality parameters. A composite of each genotype was prepared from wheat samples grown at nine locations across western Canada. Based on availability and grading information of wheat samples from the nine locations, a recipe was developed for the preparation of the wheat composites. All composites were graded as No.1 CWAD. Each of these variety composites was segregated into three size fractions using a Carter dockage tester (Simon-Day Ltd., Winnipeg, MB, USA) equipped with no. 6 (2.38 mm × 19.05 mm) and no. 7 (2.78 mm × 19.05 mm) slotted sieves. The segregated kernel size fractions were categorized as small-sized kernels (SK, through no.6 slotted sieve), medium-sized kernels (MK, passing no.7 but remained above no.6 slotted sieve), and large-sized kernels (LK, remained above no.7 slotted sieve).

### 2.2. Wheat Physical Properties

To accommodate the small sample size, the test weight (TWT) was measured using a 0.5 L container equipped with a cox funnel following the standard procedure described by the Canadian Grain Commission [8]. The value in gram per half liter was converted to kg per hectoliter using the test weight conversion chart for amber durum wheat. TKW was determined with an electronic seed counter (Model 750, The Old Mill Company, Savage, Maryland) using a 20 g sample of wheat of which all broken kernels were manually removed. KSD was determined on a series of slotted sieves (i.e., no. 6, 7, and 8). One hundred grams of wheat was subsampled and manually shaken for 30 s, after which the four fractions separated by the sieves were collected and weighted individually. All wheat physical tests were conducted in duplicate.

### 2.3. Standard Durum Milling Procedure

Following the mill flow previously described by Dexter et al. (1990) [9], original unsorted wheat samples were milled into semolina in duplicates of 2.3 kg lots with a four stand Allis-Chalmers laboratory mill (West Allis, WI, USA) in conjunction with a laboratory purifier. The mill room was controlled at 21 °C and 60% relative humidity. Semolina is defined as having less than 3% pass through a 149 μm sieve. The total milling yield is the combination of semolina and flour. Both the total and semolina yields are reported as a percentage of the cleaned wheat on a constant moisture basis. Semolina granules were prepared by adding the most refined flour stream(s) to semolina until 70% extraction was reached for quality analysis.

### 2.4. Micro-Milling and Purification Protocol

Wheat samples of various size fractions were milled to predict semolina and total milling yields following the micro-milling procedure previously developed by Wang et al. (2019) [10]. After tempering to a moisture content of 16% overnight, 200 g of wheat sample was ground with a Quadruma Junior (QJ)-II-G mill-semolina version (C.W. Brabender Instruments, Inc., South Hackensack, NJ, USA) with the original sifter removed. The resulting wholemeal was sifted through a universal laboratory sifter (Bühler MLUA GM sieve, Bühler AG) equipped with a bottom screen of 180 μm to remove the flour and a top screen of 630 μm to retain the bran-rich fraction. The unpurified semolina fraction (SY1) between the two screens was collected. Based on the prediction models developed by Wang et al. (2019) [10], semolina yield and total milling yield were calculated according to the amount of SY1 and bran-rich fraction. Formula (1) and (2) are as follows:

Semolina Yield (%) = 1.02 × Bran-rich fraction + 1.80 × SY1 − 73.17.(1)

Total Milling Yield (%) = 0.62 × SY1 + 39.42(2)

**Figure 1:**
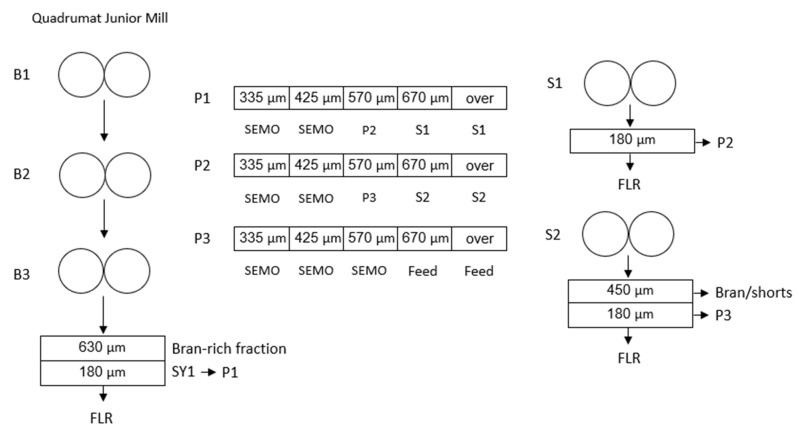
Durum micro-milling flow and purification procedure. B = break passage, FLR = flour, P = purifier, S = sizing passage, SEMO = semolina, SY1: unpurified semolina.

To prepare refined semolina for analysis and pasta processing, the original purification steps described by Dexter et al. [9] were modified to accommodate the small semolina sample size with three purification and two sizing passages. A detailed description of the micro-milling and purification steps is illustrated in Figure 1. In a typical experiment, SY1 obtained from QJ semolina mill was passed over a laboratory purifier (Namad, Rome, Italy) equipped with four different sizing sieves (335, 425, 570, and 670µm). After the first purification (P1), large semolina granules collected in tray 4 and 5 were reduced with the first sizing roll (S1). The reduced semolina was sifted through a box sifter equipped with a 180 μm sieve for 30 s to remove the flour. The resulting fraction retained above the 180 μm sieve together with the semolina collected in tray 3 at P1 were subject to a second purification (P2). After P2, the semolina granules which remained in tray 4 and 5 were subject to a second sizing step (S2). The reduced fraction was sifted with a box sifter for 30 s to remove bran/shorts (>425 μm) and flour (<180 μm). The semolina fraction between 180 and 425 μm was combined with the semolina collected in tray 3 at P2 and transferred to the third purification (P3). Refined semolina was collected as tray 1 and 2 in P1, tray 1 and 2 in P2 and tray 1, 2, and 3 in P3. Tray 4 and 5 in P3 were defined as Feeds.

### 2.5. Semolina Quality Testing

The protein content of the whole wheat and semolina were measured following the method previously described by Williams et al. [11] with a LECO Truspec N CNA (combustion nitrogen analysis) analyzer (Saint Joseph, MI). Ground wheat meal was prepared using a Retsch ZM 200 mill (Retsch GmbH, Haan, Germany) equipped with a 0.5 mm screen (Trapezoid holes) at a speed of 14,000 rpm. Ash content, wet gluten, and gluten index were determined using AACC International approved methods 76-31.01 and 38-12.02, respectively [12]. Semolina color was measured with a Minolta colorimeter CR-410 (Konica Minolta Sensing, Inc., Tokyo, Japan) with a D65 illuminant. Color readings are expressed on the CIELAB color space system with L*, a* and b* parameters representing brightness, redness, and yellowness values, respectively. A micro scale rapid extraction procedure as described by Fu et al. [13] was used for the determination of the total yellow pigment (TYP) content of the semolina.

### 2.6. Spaghetti Processing and Color Measurement

Spaghetti were produced from semolina using a customized micro-extruder (Randcastle Extrusion Systems Inc., Cedar Grove, NJ, USA) following the method of Fu et al. [6]. Semolina was first mixed with water in a high-speed asymmetric centrifugal mixer (DAC 400 FVZ SpeedMixer, FlackTec, Landum, SC, USA) at water absorption of 31–32% to maintain a constant extrusion pressure of about 100 psi. Vacuum was applied to eliminate introduction of air bubbles and minimize oxidative degradation of the yellow pigment, after which the dough crumbs were extruded through a four-hole Teflon coated spaghetti die (1.8 mm). The fresh pasta was subsequently dried in a pilot pasta dryer (Bühler, Uzwil, Switzerland) coupled with a 325 min drying cycle and a maximum temperature of 85 °C. To measure spaghetti color, 6.5 cm bands of spaghetti strands were mounted on a white mat board with minimum interspace. Spaghetti color was determined using a Minolta colorimeter (CR-410) as described above.

### 2.7. Statistical Analysis

All data were analyzed with Microsoft Excel and SAS 9.4 Software (SAS Institute Inc., Gary, NC, USA). A 3 × 7 factorial experiment was applied to evaluate the impact of kernel size and genotype on key durum wheat quality characteristics by including 3 levels of kernel size (small, medium, and large) and 7 different genotypes (A to G) representing the major source of variations. Each segregated kernel size fraction from a selected genotype was treated as an independent sample. Significance of each factor as indicated by *F* values and percentage of variability assignable to each factor as measured by the ratio of sum of square to the total sum of squares was calculated. Tukey’s test, which followed the analysis of variance, indicated significant differences with a level of *p* < 0.05.

## 3. Results and Discussion

### 3.1. Influence of Kernel Size and Genotype on Physical Properties of Durum Wheat

To understand the impact of kernel size, genotype, and their interactions on major durum wheat quality parameters, seven durum genotypes with variation in milling and color related quality attributes were segregated into three kernel size fractions using a Carter dockage tester. The wheat and semolina quality parameters of the unsorted samples are summarized in Table 1. The selected genotypes differed greatly in semolina and total milling yields, TYP, and gluten index, but with less variation in wheat physical properties (i.e., HVK, TWT, TKW, KSD), wheat protein, and ash contents. The semolina and total milling yields from the micro-milling procedure were comparable to those of standard laboratory milling except genotype D which showed higher semolina and total milling yields in the micro-milling process.

The significance of kernel size, genotype, and their interactions on major durum wheat quality parameters, as measured by the *F* value and percentage of variability assignable to each factor and their interactions, are summarized in Table 2. Significant impact was found for kernel size, genotype, and their interactions on all wheat quality parameters examined (*p* < 0.001). In terms of wheat physical properties, kernel size accounted for more than 80% of the variability in TWT and TKW with minor influences shown for genotypes and their interactions. Table 3 summarizes the impact of genotype on key quality parameters in relation to kernel size. TKW reduced drastically from 51.0 ± 1.8 g of LK to 36.1 ± 0.9 g of MK, but was only accompanied by a small decrease of TWT from 83.7 ± 0.7 kg/hL to 82.2 ± 0.6 kg/hL. Further decrease of kernel size from MK to SK led to a much greater reduction in average TWT from 82.2 kg/hL to 77.6 kg/hL, suggesting SK (TKW of 23.9 ± 0.4 g) was much less dense than the corresponding larger ones. A similar decrease in TKW and TWT was reported when a bulk CWAD cargo aggregate was fractioned into five different kernel sizes [5]. Wang and Fu reported that TWT is less effective than TKW in distinguishing the difference in kernel size [5].

Interestingly, the impact of genotype on TWT was greater for SK than for both MK and LK (Table 3). Although there was no significant difference in TKW of the SK fractions, SK possessed much greater variability in TWT, ranging from 74.5 to 80.6 kg/hL (*F* value = 465.6, *p* < 0.001) as compared to MK (81.1–82.6 kg/hL, *F* value = 73.3, *p* < 0.001) and LK (82.3 to 84.5 kg/hL, *F* value = 130, *p* < 0.001). On the other hand, greater variation in TKW among genotypes was shown for LK (48.3 to 52.8 g, *F* value = 23.81, *p* < 0.001) in comparison to MK (34.8–37.0 g, *F* value = 4.5, *p* < 0.05) and SK (23.2 to 24.4 g, *F* value = 1.9, ns).

TWT can be affected by wheat moisture, kernel density, kernel shape, and packing factors, which were not directly associated with milling yield [14,15,16,17,18]. Simmons and Meredith attributed the difference in TWT to bran surface roughness, distribution of kernel size, shape, volume, and kernel density [19]. Troccoli and di Fonzo found that kernel shape such as rectangular aspect ratio (kernel width/kernel length) and circularity shape factor (4π × area/perimeter^2^) were positively related to TWT [20]. More recently, Wang and Fu reported that durum wheat with a high proportion of SK could exhibit TWT comparable to the wheat samples of larger kernel size but exhibited much lower milling yields [5]. The relationship appears to be genotype dependent. The great variation in TWT of the SK fraction could likely be attributable to large differences in kernel shape and packing density. Due to the potential strong impact of genotype, TWT can vary widely for small-sized kernels. Therefore, TWT may not be reliable as a direct indicator of the milling potential of durum wheat when SK is predominantly present. It is critical to monitor the KSD when a larger proportion of SK is present. Wang and Fu (2020) demonstrated that by accounting for the difference in KSD, greater relationships were found for TKW (R^2^ > 0.91, *p* < 0.001) or the proportion of kernels passing the no.6 slotted sieve with milling yields than TWT alone (R^2^ = 0.75, *p* < 0.001) by studying 21 wheat composites of four major CWAD varieties [5].

### 3.2. Influence of Kernel Size and Genotype on Milling Quality of Durum Wheat

**Figure 2:**
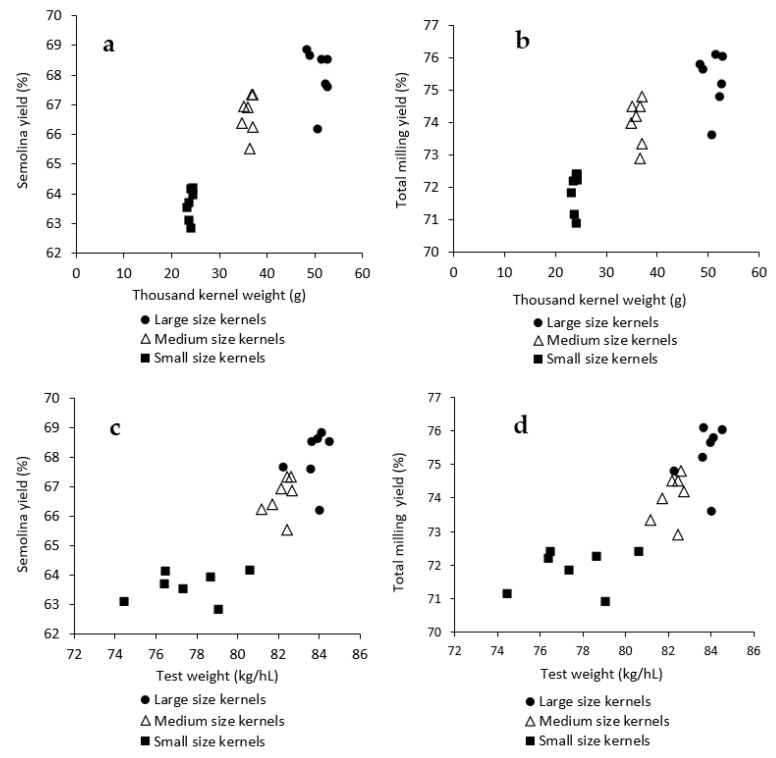
Impact of genotype and kernel size on semolina and total milling yield of selected durum samples in relation to thousand kernel weight (**a**,**b**) and test weight (**c**,**d**).

From Table 2, a significant impact of kernel size, genotype and their interactions was found on durum milling performance (semolina and total milling yields and semolina ash content). Based on the ANOVA test, more than 80% of variation in milling yields was attributed to kernel size alone, with a greater impact of kernel size being noted for semolina yield than total milling yield (*F* value: 13177.7 vs. 7392.8). Figure 2 demonstrates the semolina and total milling yields in relation to TKW and TWT as affected by kernel size. Regardless of genotype selected, decrease of kernel size significantly reduced semolina and total milling yields. A drastic reduction of milling yields was evident for kernels passing no.6 slotted sieve (Table 3). On average, LK (68.0 ± 0.9%) had 1.3% higher SY than MK (66.7 ± 0.7%), and the latter was about 3.1% higher in SY than that of SK (63.6 ± 0.7%). Kernel size is clearly a better indicator of average milling yields for SK than the TWT (Figure 2). For LK and MK; however, both TWT and TKW provided strong indication of average milling quality. A similar adverse effect of SK on durum milling quality was reported by Wang and Fu (2020) and Dexter et al. (2007) by examining durum composites with a wide variation in kernel sizes [4,5].

From Figure 2, considering the response of genotype to the relationship between kernel size and milling quality, genotypes A and B appeared to be more susceptible to kernel size variations showing a greater decrease (~4.9%) in semolina yield from 68.9 to 64.0% than those of the inferior ones (e.g., G) from 66.2 to 62.9% (vs. 3.3%). A similar trend was found for total milling yield (3.5% vs. 2.7%). There were significant differences in semolina and total milling yields among the genotypes at all three kernel size fractions (Figure 2a,b). The difference in milling yields was greater for LK (2.7%) than MK (1.8%) and SK (1.3%) among the selected genotypes (Table 3).

When comparing milling quality of all kernel size fractions (Figure 2), semolina and total milling yields of MK segregated from genotypes with superior milling quality (A and C) were comparable or superior to the LK from genotypes of inferior or moderate milling quality (E, F, and G) despite the TKW of those MK (34.8 to 37.0 g) being significantly lower than LK counterparts (48.3 to 52.8 g). In addition, LK from genotypes with inferior milling quality showed lower milling yields. SK exhibited inferior milling quality to both MK and LK regardless of the genotypes selected (Table 3). SK is very detrimental to the overall milling quality but usually represents only a small proportion in commercial durum shipments. Analysis of variance by excluding SK revealed that genotype accounted for 52.0% of variation in semolina yield, followed by kernel size of 44.3% and their interaction of 3.4%. These results strongly suggest that the intrinsic kernel properties could play an important role in determining the milling quality of durum wheat. Selection of genotypes with superior milling quality could compensate the negative impact of SK which is usually present in higher percentage in dry and hot growing seasons. When a large proportion of small kernels was present; however, milling quality could be poor regardless of the genotypes selected.

In addition to the milling yields, ash content is an important part of overall milling quality. The ash contents of wheat and semolina increased with the decrease of kernel size (Table 3). Coupled with the lower semolina yield of SK, its high semolina ash could further decrease the wheat milling potential when a constant degree of semolina refinement is required.

Milling quality of durum wheat is a complicated trait [10]. From Figure 2, a cooperative effect between kernel size and genotype on durum milling quality was evident when considering both MK and LK. The average milling yields of SK were lower and the impact of genotype was much less (Table 3). While the impact of some common kernel physical parameters (e.g., vitreousness, TWT, and KSD) on milling quality has been extensively investigated, the work on the intrinsic properties that contribute to varietal differences in milling quality of durum wheat are scarce [19,21,22,23,24]. Both kernel morphological parameters (e.g., length, width, thickness, size, shape, etc.) and kernel physical properties (e.g., hardness, vitreousness, TWT) could affect milling quality. Simmons and Meredith (1979) summarized three major factors that contribute to the difference in milling quality: the amount of endosperm contained in the grain (endosperm-to-bran ratio); the separability of the endosperm from the aleurone and bran layers (structure dissociates on fracture and milling); and endosperm hardness, which determines how the kernel fragments during the milling process [19]. Novaro et al. (2001) reported ellipsoidal volume was the best predictor of semolina yield among other grain morphological parameters evaluated [25]. Haraszi et al. found that the rheological phenotype phases of an average crush response profile obtained from a single kernel characterization system provided good predictions of the laboratory milling potential of durum wheats [26].

Due to the relatively large kernel size of the original unsorted samples (Table 1) and the similar TKW of the segregated kernel fractions (Table 3), the varietal differences in milling quality among selected genotypes could be attributed to their intrinsic kernel properties. Information on hardness, endosperm-to-bran ratio, and kernel fracture behavior could shed some light on the genotypic variation in milling quality. A study is currently being conducted in our laboratory to investigate the underlying factors, which could affect the milling quality of durum genotypes with a similar size of wheat kernels.

### 3.3. Influence of Kernel Size and Genotype on Semolina and Pasta Color Parameters

**Figure 3:**
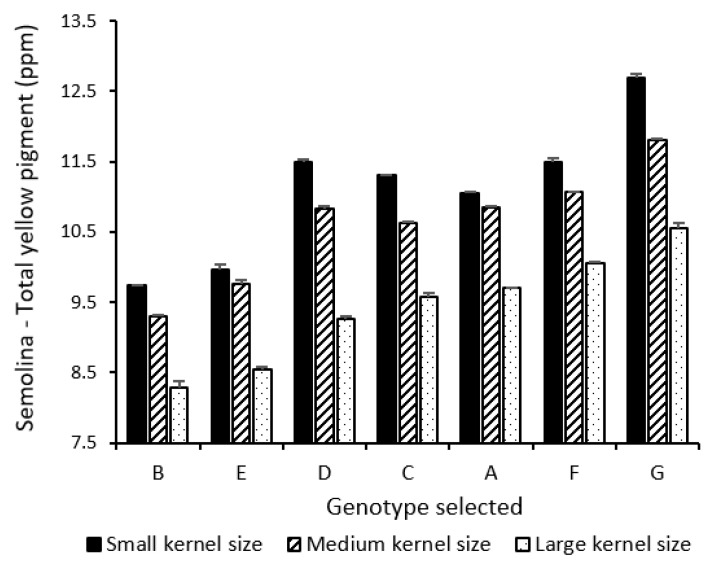
Impact of genotype and kernel size on semolina total yellow pigment content of selected genotypes.

Both genotype and kernel size significantly affected semolina TYP (Table 2). Figure 3 presents the semolina TYP of three kernel size fractions segregated from the selected genotypes. The decrease of kernel size led to significant increase in semolina TYP for all genotypes. Alvarez et al. (1999) reported a similar negative relationship between kernel weight and yellow pigment concentration [27]. A greater difference in TYP was shown between MK and LK (1.0–1.6 ppm) than between small and medium ones (0.2–0.9 ppm). The degree of increase in semolina TYP as shown in Figure 3 was comparable to the level previously reported by Wang and Fu, who found that semolina TYP of SK was about 1.5 ppm higher than that of LK segregated from a bulk CWAD cargo composite [5]. Large genetic variations in semolina TYP from 2.3 to 3.0 ppm were noted for the genotypes used in this study across three different kernel sizes.

**Figure 4:**
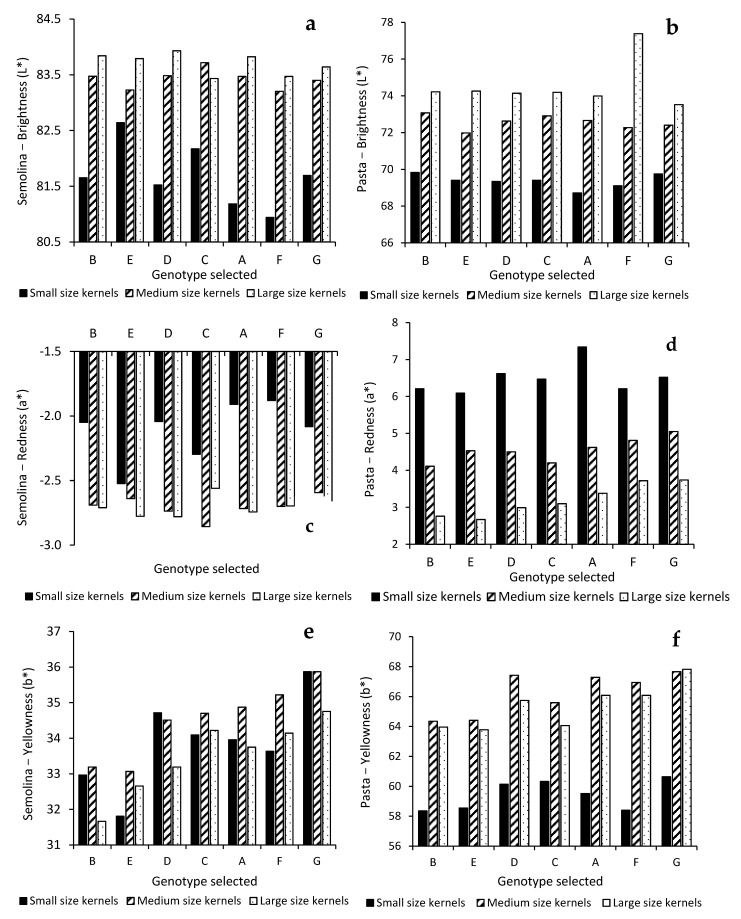
Impact of genotype and kernel size on semolina (**a**,**c**,**e**) and pasta color (**b**,**d**,**f**).

The colour of semolina and pasta made from the size fractions are summarized in Figure 4. Brightness and redness of semolina were greatly influenced by kernel size, while the genotype had a large impact on semolina yellowness (Table 2). In general, semolina prepared from MK and LK was much brighter (Figure 4a) and less dull (Figure 4c) compared to that prepared from SK. Much greater variation in brightness and redness was also shown for SK fractions than MK and LK ones (Figure 2 and Table 3).

With the decrease of kernel size from LK to MK, significant increases in semolina TYP and yellowness were shown (Figure 4e). However, except for genotypes D and G, reduction of kernel size from MK to SK did not lead to further increase in semolina yellowness despite the TYP being significantly higher in SK. The drastic decrease in semolina brightness and increase in redness for small kernels might mask semolina yellowness.

**Figure 5:**
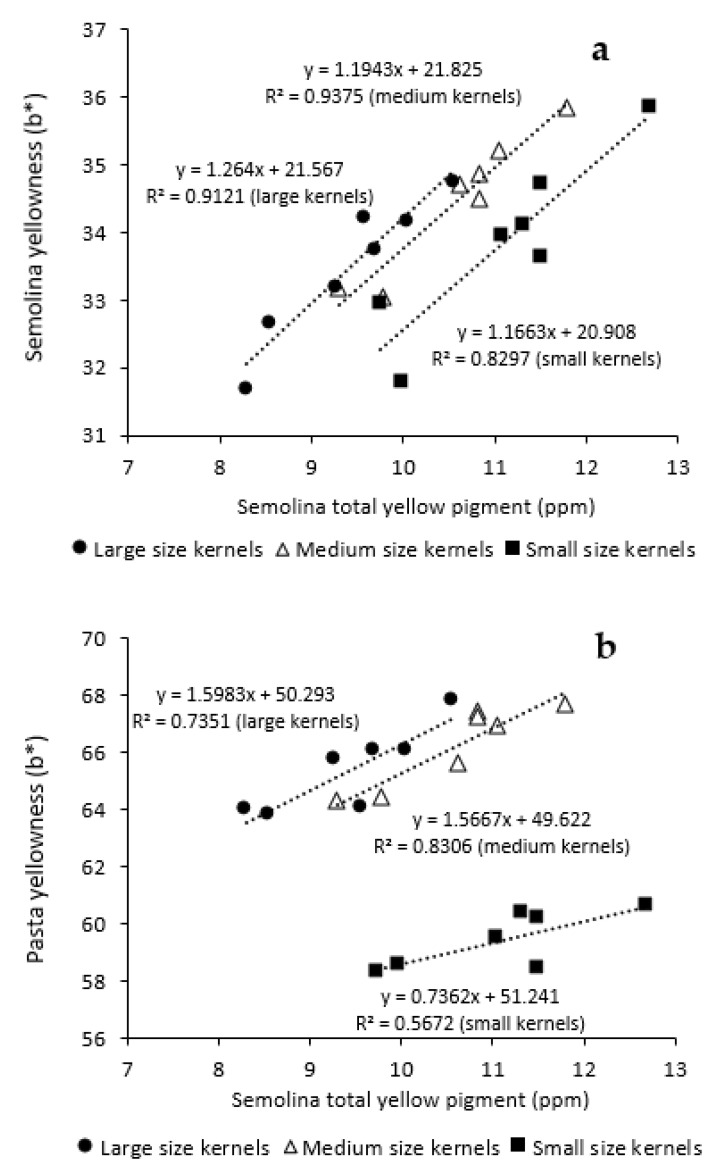
Combined effect of kernel size and genotype on yellowness of semolina (**a**) and pasta (**b**).

Table 2 showed a large impact of kernel size on pasta color. The decrease in kernel size led to a significant reduction in pasta brightness (Figure 4b) and an increase in pasta redness (Figure 4d). Superior yellowness was seen for pasta prepared from medium and large kernel fractions. However, a drastic decrease in pasta yellowness of about 7 units was noticed for SK despite its semolina TYP being significantly higher (Figure 4f). By plotting semolina yellowness against TYP for three different kernel size fractions of the selected genotypes, it was shown that semolina b* linearly increased about 1.2 units with each ppm increase in TYP (Figure 5a). The degree of increase in semolina yellowness in relation to TYP was similar for all three size fractions. For a given TYP, however, semolina prepared from LK and MK consistently showed superior yellowness than that of SK, inferring the negative impact of SK on semolina yellowness. This negative impact was much more profound for pasta yellowness (Figure 5b). As far as SK fraction is concerned, the increase in semolina TYP resulted in little increase in pasta yellowness. This is in contrast to the MK and LK fractions evaluated in this study.

Pasta brightness and yellowness decrease with the increase of semolina ash content [28,29]. Although SK have lower semolina and total milling yields, the higher ash content suggests inclusion of a greater proportion of external tissues, which could lead to pasta browning due to high enzymatic activities [28]. Maillard reaction between amino acid and reducing sugars could lead to the undesirable reddish color of pasta dried at high temperature [30,31]. Although the protein content was not significantly higher for SK as compared with MK and LK, pasta prepared from SK was much redder (6.2–7.3 in a*) than that made from LK (2.7–3.7 in a*), suggesting other underlying factors such as amino acid composition or reducing sugar content may favor the development of the reddish coloration of pasta prepared from small kernels. Joubert et al. revisited the role of particle size, ash, and protein on pasta color and viscoelasticity [32]. By combining the milling fractions of five durum wheat patches, a series of formulated mixes of semolina/flour were prepared so that the effect of protein, ash, and particle size distribution (PSD) could be evaluated in an unbiased manner. The authors found that pasta brightness and yellowness decreased while redness increased with the increase of semolina ash content regardless of protein content and PSD. The authors attributed the increase in pasta redness to the elevation of reducing sugars accompanied by the high ash content in the semolina. A significant correlation was found between the ash content and total arabinoxylans in semolina, which were known to concentrate in the outer layers of the grain [33]. The extrusion process can significantly increase the reducing sugars due to shearing stress [34]. It is likely that the SK contains a high level of arabinoxylan, which could result in a high level of reducing sugar during extrusion and increase the potential of Maillard reactions [32]. The elevated redness/brownness and decrease in pasta brightness could subsequently mask pasta yellowness. Wang and Fu proposed that the drastic elevation in pasta redness due to the Maillard reaction under high-temperature (85 °C) drying conditions could adversely impact pasta yellowness regardless of the level of TYP [5].

## 4. Conclusions

By segregating durum samples of selected genotypes into three kernel size fractions, the impact and relative importance of kernel size, genotype, and their interaction on major quality parameters were characterized in this study. For LK and MK fractions, TWT and kernel size are closely related. However, a greater influence of genotype on TWT of SK was evident. Regardless of the genotype, the SK fraction is detrimental to durum milling performance as shown by low semolina yield, high semolina ash content, and poor semolina color. The degree of impact of genotype on the durum milling performance appears to be related to kernel size. A greater impact was shown for LK than MK and SK, based on seven genotypes evaluated in this study. When the SK fraction is excluded, the genotype or intrinsic property of the durum kernel played an important role in contributing to overall milling quality. Genotype is a dominant factor in determining semolina TYP and yellowness despite TYP increases with the decrease of kernel size. Semolina and pasta prepared from MK and LK fractions were much brighter and less dull than those made from SK. Regardless of the genotype, the SK fraction exerted a strong detrimental effect on pasta yellowness, despite the higher level of TYP in SK. To meet the milling and end-product quality expectation of domestic and international durum buyers, it is critical to monitor the presence of SK (through a no.6 slotted sieve) in commercial durum samples, particularly in hot and dry growing seasons. More research is needed to confirm the potential interactions between genotype and kernel size and their effects on durum quality by using wheat samples from various genotypes and different growing conditions.

## Figures and Tables

**Table 1:** Wheat and semolina quality parameters of selected genotypes.

Line	Wheat Properties									Standard Milling		Micro-Milling		Semolina Properties-Standard Milling			
	Grade	HVK (%)	TWT (kg/hL)	TKW (g)	KSD (%)			Protein (%)	Ash (%)	SY (%)	TMY (%)	SY (%)	TMY (%)	Protein (%)	Ash (%)	TYP (ppm)	Gluten index(%)
					Small	Medium	Large										
A	1	94	83.6	48.0	5.8	22.3	71.9	14.3	1.40	68.1	76.2	68.1	75.7	13.3	0.65	10.3	62
B	1	92	83.1	44.8	5.8	29.3	64.9	14.5	1.37	68.0	76.0	68.0	75.1	13.4	0.65	9.0	70
C	1	92	82.8	45.7	6.0	24.6	69.4	15.2	1.38	67.2	75.3	67.8	75.6	13.1	0.61	10.7	85
D	1	91	82.8	43.2	7.6	33.2	59.2	14.3	1.47	66.3	74.7	67.8	75.0	14.2	0.62	10.3	77
E	1	95	83.0	46.6	4.9	26.3	68.9	14.9	1.39	66.9	75.6	67.0	74.7	13.9	0.62	9.4	66
F	1	93	81.5	47.7	3.5	22.4	74.2	15.5	1.45	66.1	73.4	67.0	74.2	14.4	0.63	10.8	57
G	1	96	83.0	44.7	7.4	32.3	60.3	14.7	1.44	65.2	73.4	65.7	73.4	13.7	0.69	11.5	56
Mean		93.3	82.8	45.8	5.9	27.2	67.0	14.8	1.42	66.8	74.9	67.3	74.8	13.8	0.64	10.3	69
Range		5	2.1	4.8	4.1	10.9	15.0	1.2	0.10	2.9	2.8	2.4	2.3	1.3	0.08	2.5	29

Note: small kernels: through No.6 slotted sieve (2.38 × 19.05 mm); medium kernels: passing No.7 but remained above No.6 slotted sieve; large kernels: remained above No.7 slotted sieve (2.78 × 19.05 mm). HVK = hard vitreous kernels; TWT = test weight; TKW = thousand kernel weight; KSD = kernel size distribution; SY = semolina yield; TMY = total milling yield; TYP = total yellow pigment.

**Table 2:** Significance of kernel size, genotype, and their interaction on major durum wheat quality parameters as measured by F values and percentage of variability assignable to each factor and their interaction.

	*F* Values			Percentage of Variability Assignable to Each Factor			
	Kernel Size	Genotype	Interactions	Kernel Size	Genotype	Interactions	Error
Wheat properties							
TWT	502 ****	215.2 ****	66.8 ****	82.6	10.6	6.6	0.2
TKW	9365.2 ****	8.3 ***	11.2 ****	98.9	0.3	0.7	0.1
Protein	555.5 ****	2159.1 ****	68.9 ****	7.4	86.6	5.5	0.4
Ash	800.9 ****	281.2 ****	33.9 ****	42.6	44.9	10.8	1.7
Milling quality							
Semolina yield	13177.7 ****	546.9 ****	36.0 ****	87.6	10.9	1.4	0.1
Total milling yield	7392.8 ****	531.9 ****	24.4 ****	80.8	17.4	1.6	0.1
Semolina ash	2143.3 ****	131.0 ****	30.6 ****	77.9	14.3	6.7	1.1
Semolina color							
Semolina TYP	2897.9 ****	1276.6 ****	24.5 ****	42.1	55.6	2.1	0.2
L*	608.8 ****	10.4 ****	9.6 ****	84.7	4.3	8.0	2.9
a*	238.2 ****	5.7 ***	6.6 ****	75.5	5.4	12.5	6.7
b*	96.0 ****	177.8 ****	13.5 ****	13.1	73.0	11.0	2.9
Pasta color							
L*	18998 ****	184.9 ****	293.1 ****	89.1	2.6	8.1	0.1
a*	30824.8 ****	452.5 ****	150.0 ****	93.1	4.0	2.7	0.1
b*	9625.5 ****	406.4 ****	59.0 ****	85.9	10.9	3.2	0.1

***, **** indicate significance level of 0.001 and 0.0001. TWT = test weight; TKW = thousand kernel weight; TYP = total yellow pigment.

**Table 3:** Impact of genotype on wheat, milling, semolina, and pasta quality parameters of selected genotypes at three different level of kernel sizes.

	Wheat Properties				Milling Quality			Semolina Color				Pasta Color		
	TWT (kg/hL)	TKW (g)	Protein (%)	Ash (%)	SY (%)	TMY (%)	Semolina Ash (%)	Semolina TYP	L*	a*	b*	L*	a*	b*
Large kernels														
Mean	83.7	51.0	14.8	1.41	68.0	75.3	0.57	9.4	83.7	−2.7	33.5	74.5	3.2	65.4
SD	0.7	1.8	0.5	0.04	0.9	0.9	0.02	0.8	0.2	0.1	1.1	1.3	0.4	1.5
Range	2.2	4.5	1.3	0.11	2.7	2.5	0.06	2.3	0.5	0.2	3.1	3.9	1.1	4.0
*F* values	130 ****	23.81 ***	1343.5 ****	130.6 ****	203.7 ****	220.4 ****	52. 7****	511 ****	9.27 ***	5.7 **	49.5 ***	658 ****	299 ****	223 ****
Medium kernels														
Average	82.2	36.1	14.6	1.41	66.7	74.0	0.60	10.6	83.4	−2.7	34.5	72.6	4.6	66.2
SD	0.6	0.9	0.5	0.04	0.7	0.7	0.02	0.8	0.2	0.1	1.0	0.4	0.3	1.4
Range	1.5	2.2	1.3	0.08	1.8	1.9	0.05	2.5	0.5	0.3	2.8	1.1	0.9	3.3
*F* values	73.3 ****	4.5 *	1100.1 ****	96.0 ****	170.6 ****	160.8 ****	73.2 ****	1464 ****	11.5 ***	11.5 ***	68 ***	56 ****	171 ****	205 ****
Small kernels														
Average	77.6	23.9	14.9	1.49	63.6	71.9	0.67	11.1	81.7	−2.1	33.9	69.4	6.5	59.4
SD	2.0	0.4	0.4	0.05	0.5	0.6	0.03	1.0	0.6	0.2	1.3	0.4	0.4	1.0
Range	6.1	1.2	1.2	0.15	1.3	1.5	0.07	3.0	1.7	0.6	4.1	1.1	1.3	2.3
*F* values	465.6 ****	1.9 ns	386.4 ****	121.0 ****	128.6 ****	106.1 ****	70.3 ****	2149 ****	17.3 ****	9 ***	92 ***	57 ****	282 ****	96 ****

*, **, ***, **** indicate significance level of 0.05, 0.01, 0.001 and 0.0001, respectively. ns = not significant; SD = standard deviation; TWT = test weight; TKW = thousand kernel weight; SY = semolina yield; TMY = total milling yield; TYP = total yellow pigment.

## References

[1] Feillet P., Dexter J.E., Kruger J.E., Matsuo R.B., Dick J.W. (1996). Quality requirements of durum wheat for semolina milling and pasta production. Pasta and Noodle Technology.

[2] Sissions M., Abecassis J., Marchylo B., Sissions M., Abecassis J., Marchylo B., Carcea M. (2012). Methods used to access and predict quality of durum wheat, semolina, and pasta. Durum Wheat Chemistry and Technology.

[3] Dexter J.E., Matsuo R.R., Martin D.G. (1987). The relationship of durum wheat test weight to milling performance and spaghetti quality. CFW.

[4] Dexter J.E., Symons S.J. (2007). Impact of durum wheat test weight, kernel size, kernel weight and protein content on semolina milling quality. Int. Miller..

[5] Wang K., Fu B.X. (2020). Inter-relationships between test weight, thousand kernel weight, kernel size distribution and their effects on durum wheat milling, semolina composition and pasta processing quality. Foods.

[6] Fu B., Wang K., Dupuis B., Taylor D., Nam S. (2017). Kernel vitreousness and protein content: Relationship, interaction and synergistic effects on durum wheat quality. J. Cereal Sci..

[7] Baasandorj T., Ohm J., Manthey F., Simek S. (2015). Effect of kernel size and mill type on protein, milling yield, and baking quality of hard red spring wheat. Cereal Chem..

[8] Canadian Grain Commission: Procedure and Equipment for Determining Test Weight. https://www.grainscanada.gc.ca/en/grain-quality/grain-grading/grading-factors/grading-factors-wheat/test-weight.html.

[9] Dexter J.E., Matsuo R.R., Kruger J.R. (1990). The spaghetti making quality of commercial durum wheat samples of variable amylase activity. Cereal Chem..

[10] Wang K., Taylor D., Pozniak C., Fu B.X. (2019). Developing a high-throughput micromilling protocol for evaluating durum wheat milling performance and semolina quality. Cereal Chem..

[11] Williams P., Sobering D., Antoniszyn J., Fowler D.B., Geddes W.E., Johnston A.M., Preston K.R. (1998). Protein testing methods. Wheat Protein Production and Marketing.

[12] AACC International (2010). Approved Method of Analysis.

[13] Fu B., Schlichting L., Pozniak C.J., Singh A.K. (2013). Pigment loss from semolina to dough: Rapid measurement and relationship with pasta color. J. Cereal Sci..

[14] Hook S.C. (1984). Specific weight and wheat quality. J. Sci. Food Agric..

[15] Tkachuk R., Kuzina F.D. (1979). Wheat: Relationship between some physical and chemical properties. Can. J. Plant Sci..

[16] Cabral A.L., Jordan M.C., Larson G., Somers D.J., Humphreys D.G., McCartney C.A. (2018). Relationships between QTL for grain shape, grain weight, test weight, milling yield, and plant height in the spring wheat cross RL4452/’AC Domain’. PLoS ONE.

[17] Yabwalo D.N., Berzonsky W.A., Brabec D., Pearson T., Glover K.D., Kleinjan J.L. (2018). Impact of grain morphology and genotype by environment interactions on test weight of spring and winter wheat (*Triticum aestivum* L.). Euphytica.

[18] Samaan J., EI-Khayat G.H., Manthey F.A., Fuller M.P., Brennan C.S. (2006). Durum wheat quality: II. The relationship of kernel physicochemical composition to semolina quality and end product utilization. Int. J. Food Sci. Technol..

[19] Simmons L., Meredith P. (1979). Width, weight, endosperm, and bran of the wheat grain as determinants of flour milling yield in normal and shrivelled wheats. N. Z. J. Sci..

[20] Troccoli A., di Fonzo N. (1999). Relationship between kernel size features and test weight in Triticum durum. Cereal Chem..

[21] Dziki D., Laskowski J. (2005). Wheat kernel physical properties and milling process. Acta Agrophysica.

[22] Lyford C.P., Kidd W., Rayas-Duarte P., Deyoe C. (2005). Prediction of flour extraction rate in hard red winter wheat using the single kernel characterization. J. Food Qual..

[23] Chaurand M., Lempereur I., Roulland T.M., Autran J.C., Abecassis J. (1999). Genetic and agronomic effects on semolina milling value of durum wheat (*Triticum durum* Desf.). Crop. Sci..

[24] Peyron S., Surget A., Mabille F., Autran J.C., Rouau X., Abecassis J. (2002). Evaluation of tissue dissociation of durum wheat grain (*Triticum durum* Desf.) generated by milling process. J. Cereal Sci..

[25] Novaro P., Colucci F., Venora G., D’Egidio M.G.D. (2001). Image analysis of whole grains: A noninvasive method to predict semolina yield in durum wheat. Cereal Chem..

[26] Haraszi R., Sissons M., Juhasz A., Kadkol G., Tamas L., Anderssen R. (2016). Using rheological phenotype phases to predict rheological features of wheat hardness and milling potential of durum wheat. Cereal Chem..

[27] Alvarez J.B., Martin L.M., Martin A. (1999). Genetic variation for carotenoid pigment content in the amphiploid *Hordeum chilense x Triticum turgidum* conv. durum. Plant Breed..

[28] Borrelli G.M., Troccoli A., Di Fonzo N., Fares C. (1999). Durum wheat lipoxygenase activity and other quality parameters that affect pasta color. Cereal Chem..

[29] Matsuo R.R., Dexter J.E. (1980). Relationship between some durum wheat physical characteristics and semolina milling properties. Can. J. Plant Sci..

[30] Acquistucci R. (2000). Influence of Maillard reaction on protein modification and color development in pasta. Comparison of different drying conditions. LWT.

[31] Anese M., Nicoli M.C., Massini R., Lerici C.R. (1999). Effects of drying processing on the Maillard reaction in pasta. Food Res. Int..

[32] Joubert M., Morel M., Lullien-Pellerin V. (2018). Pasta color and viscoelastivity: Revisiting the role of particle size, ash, and protein content. Cereal Chem..

[33] Lempereur I., Rouau X., Abecassis J. (1997). Genetic and agronomic variation in arabinoxylan and ferulic acid contents of durum wheat (Triticum durum.) grain and its milling fractions. J. Cereal Sci..

[34] Lintas C., D’Appolonia B.L. (1973). Effect of spaghetti processing on semolina carbohydrates. Cereal Chem..

